# Defining Proximity Proteome of Histone Modifications by Antibody-mediated Protein A-APEX2 Labeling

**DOI:** 10.1016/j.gpb.2021.09.003

**Published:** 2021-09-30

**Authors:** Xinran Li, Jiaqi Zhou, Wenjuan Zhao, Qing Wen, Weijie Wang, Huipai Peng, Yuan Gao, Kelly J. Bouchonville, Steven M. Offer, Kuiming Chan, Zhiquan Wang, Nan Li, Haiyun Gan

**Affiliations:** 1 Shenzhen Key Laboratory of Synthetic Genomics, Guangdong Provincial Key Laboratory of Synthetic Genomics, CAS Key Laboratory of Quantitative Engineering Biology, Shenzhen Institute of Synthetic Biology, Shenzhen Institute of Advanced Technology, Chinese Academy of Sciences, Shenzhen 518055, China; 2 Cold Spring Harbor Laboratory, Cold Spring Harbor, NY 11724, USA; 3 Department of Molecular Pharmacology and Experimental Therapeutics, Mayo Clinic, Rochester, MN 55905, USA; 4 Mayo Clinic College of Medicine, Rochester, MN 55905, USA; 5 Mayo Clinic Cancer Center, Rochester, MN 55905, USA; 6 Department of Biomedical Sciences, City University of Hong Kong, Hong Kong Special Administrative Region 999077, China; 7 Key Laboratory of Biochip Technology, Biotech and Health Centre, Shenzhen Research Institute of City University of Hong Kong, Shenzhen 518172, China; 8 Division of Hematology, Department of Medicine, Mayo Clinic, Rochester, MN 55905, USA

**Keywords:** Proximity labeling, Post-translationally, AMAPEX, Modified histone

## Abstract

**Proximity labeling** catalyzed by promiscuous enzymes, such as APEX2, has emerged as a powerful approach to characterize multiprotein complexes and protein–protein interactions. However, current methods depend on the expression of exogenous fusion proteins and cannot be applied to identify proteins surrounding **post-translationally** modified proteins. To address this limitation, we developed a new method to label proximal proteins of interest by antibody-mediated protein A-ascorbate peroxidase 2 (pA-APEX2) labeling (**AMAPEX**). In this method, a modified protein is bound *in situ* by a specific antibody, which then tethers a pA-APEX2 fusion protein. Activation of APEX2 labels the nearby proteins with biotin; the biotinylated proteins are then purified using streptavidin beads and identified by mass spectrometry. We demonstrated the utility of this approach by profiling the proximal proteins of histone modifications including H3K27me3, H3K9me3, H3K4me3, H4K5ac, and H4K12ac, as well as verifying the co-localization of these identified proteins with bait proteins by published ChIP-seq analysis and nucleosome immunoprecipitation. Overall, AMAPEX is an efficient method to identify proteins that are proximal to **modified histones**.

## Introduction

Biological functions are regulated by interacting biomolecules (protein, DNA, RNA, *etc.*), and dysregulation of these interactions can lead to human diseases including cancers [1], [2]. Methods that map these interactions provide tools to study biological processes and facilitate therapy development for human diseases. Recently, proximity labeling was developed and has been shown to efficiently map molecular interactions. Proximity labeling uses engineered enzymes, such as peroxidase or biotin ligase, which are fused to a protein of interest [3] to modify its nearby associated factors. This method has been successfully utilized to map protein–protein [3], RNA–protein [4], [5], [6], protein–DNA [7], [8], and chromatin interactions [9]. However, proximity labeling has the following limitations. First, expression of exogenous fusion proteins with engineered enzymes is required, which limits its use in difficult-to-transfect cells and tissues. Second, mapping of the biomolecules that are proximal to post-translationally modified (PTM) proteins, such as histones, is challenging.

Histone modifications play critical roles in regulating basic biological processes to maintain cell identity and genome integrity [10], [11]. These modifications are recognized by reader proteins and then form functional multiprotein complexes with other regulatory factors in a spatiotemporal manner [12]. Alterations in the interacting networks of the histone modifications can lead to human diseases [13]. Therefore, systemic mapping of histone modification proximal proteins is of great importance. Current proteomics-based assays to measure the affinity between proteins and chromatin marks [14], [15], [16], [17], [18] rely on the use of synthetic histone peptides, *in vitro* reconstituted nucleosomes, or expression of external protein domains. One chromatin-context approach, chromatin-interacting protein-mass spectrometry (ChIP-MS), requires crosslinking and shearing of chromatin, thereby often resulting in high backgrounds [19]. Therefore, it is challenging to identify the proximal protein interactome of histone modifications *in situ*.

Here, we overcome the limitations of traditional proximity labeling methods using a protein A-APEX2 (pA-APEX2) fusion protein. The histone mark of interest is bound *in situ* by a specific antibody, which then tethers a pA-APEX2 fusion protein. Activated APEX2 biotinylates nearby proteins, which will then be enriched using streptavidin beads and identified using MS.

## Results

### Development of antibody-mediated pA-APEX2 labeling to identify histone modification proximal proteins *in situ*

**Figure 1:**
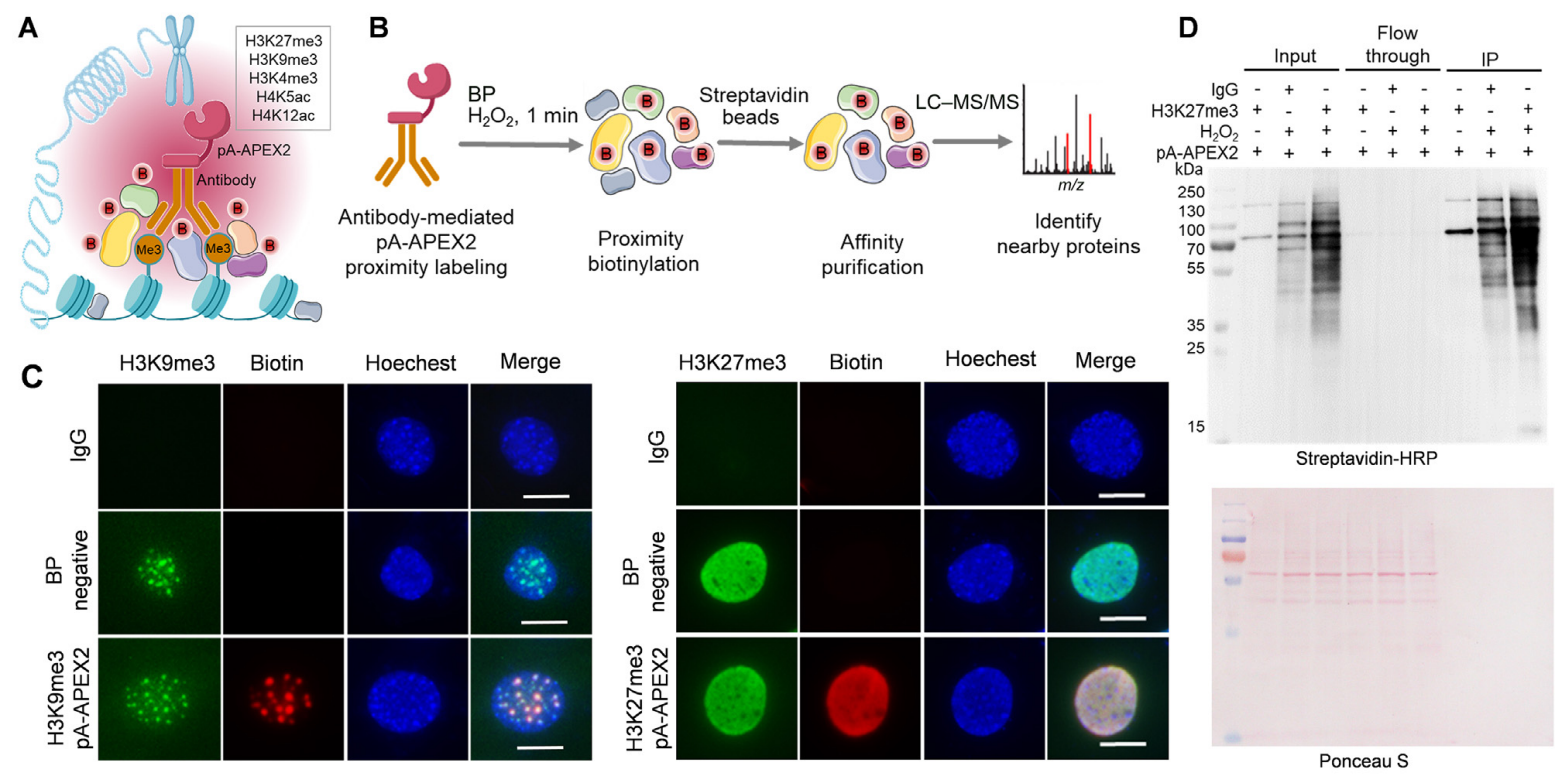
**Antibody-mediated proximity biotinylation by pA-APEX2** **A.** Illustration of antibody-mediated pA-APEX2 proximity labeling. pA-APEX2 is recruited to the targeting sites by specific histone modification antibodies. BP and H_2_O_2_ are added to cells fixed with 0.1% formaldehyde and incubated for 1 min to induce biotinylation of proteins less than 20 nm adjacent to APEX2. **B.** Biotinylated proteins are purified using streptavidin beads and analyzed by LC–MS/MS. **C.** Fluorescence imaging of histone modifications and antibody-mediated biotinylation. H3K9me3 and H3K27me3 were visualized by immunofluorescence staining (green). Biotinylation was induced as indicated before and visualized by staining with streptavidin-Cy3 (red). Nuclei were counterstained with Hoechest33342. Scale bars, 10 μm. **D.** pA-APEX2-mediated protein labeling in whole-cell lysates. Whole-cell extracts from MEF cells were incubated with pA-APEX2 and H3K27me3 antibody, and biotinylation was induced as indicated before and analyzed by Western blotting. The lower panel shows Ponceau S staining as a loading control. pA, protein A; APEX2, ascorbate peroxidase 2; BP, biotin-phenol; LC–MS/MS, liquid chromatography–tandem mass spectrometry; MEF, mouse embryonic fibroblast; IP, immunoprecipitation.

The strategy behind antibody-mediated pA-APEX2 labeling (AMAPEX) is to tether a peroxidase or biotin ligase to antibodies that are specifically bound to a protein of interest (here, histone modifications; Figure 1A). Subsequent activation of the tethered enzyme should result in biotinylation of biomolecules near the target protein. Identification of the biotinylated proteins by MS will provide information about the nearby proteomic landscape of the protein of interest (Figure 1B). We selected APEX2 as the enzyme of choice, because it has a robust enzymatic activity *in vitro* and can be stringently controlled by H_2_O_2_ [5].

**Supplementary Figure S1:**
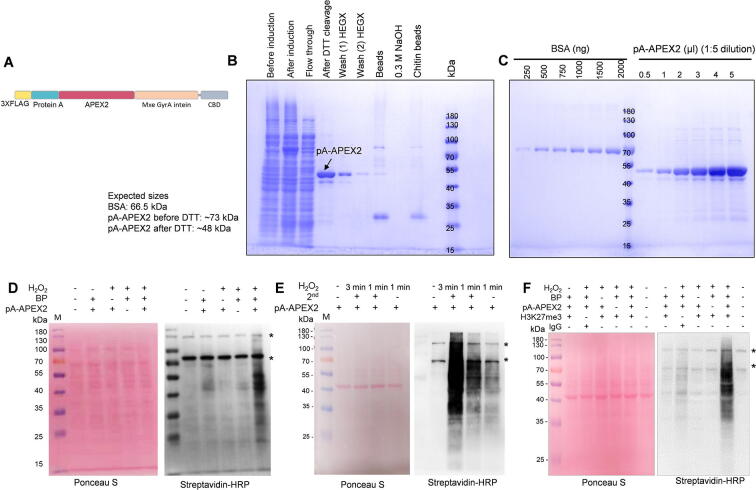
**Purified pA-APEX2 is enzymatically active toward whole-cell lysate A.** The components of pA-APEX2 fusion protein. pA, which contains two IgG binding domains of staphylococcal protein A, was fused to APEX2 peroxidase. 3×FLAG tag and Mxe GyrA intein-CBD from chitinase A1 were linked to pA-APEX2 on its N- and C-termini, respectively. **B.** Purification process of pA-APEX2. **C.** Concentration determination of pA-APEX2 by BSA standards (4 μg/μL). **D.** pA-APEX2 labeling *in vitro*. pA-APEX2 and indicated supplements for the peroxidase reaction were incubated with whole-cell lysates as indicated, and the reaction product was analyzed by Western blotting. **E.** Titration of reaction time and supplements for the pA-APEX2 reaction. **F.** Western blotting analysis of H3K27me3-mediated pA-APEX2 proximity protein labeling in living cells by change labeling time or secondary antibody. Whole-cell lysate was analyzed by Ponceau staining (left) and Western blotting (right). “*” denotes endogenous biotinylated proteins. pA, protein A segment; CBD, chitin binding domain.

We confirmed the enzymatic activity of the purified pA-APEX2 by labeling the whole-cell lysate *in vitro* (Figure S1A–F). We then tested if the enzymatically active pA-APEX2 can be recruited to the protein of interest by specific antibodies using immunofluorescence assay. The permeabilized cells were incubated with H3K9me3/H3K27me3 antibodies followed by pA-APEX2. The unbound pA-APEX2 was extensively washed out, and biotinylation was induced by adding H_2_O_2_ and biotin-phenol (BP). The co-localization of biotin and H3K9me3/H3K27me3 suggests that pA-APEX2 can be recruited to specific sites and activated in a controlled manner *in situ* (Figure 1C). As expected, no enrichment was observed in the samples without BP or in the IgG controls. We next sought to test the activity of pA-APEX2 in cell suspension. Lightly crosslinked mouse embryonic fibroblast (MEF) cells were permeabilized and incubated with H3K27me3 antibody followed by pA-APEX2 (see Materials and methods for details; File S1). Biotinylation of the proteins around H3K27me3 was induced and then analyzed by Western blotting (Figure 1D). Efficient biotin labeling in the presence of H3K27me3 antibody indicated that pA-APEX2 targeting can be accurately controlled.

**Figure 2:**
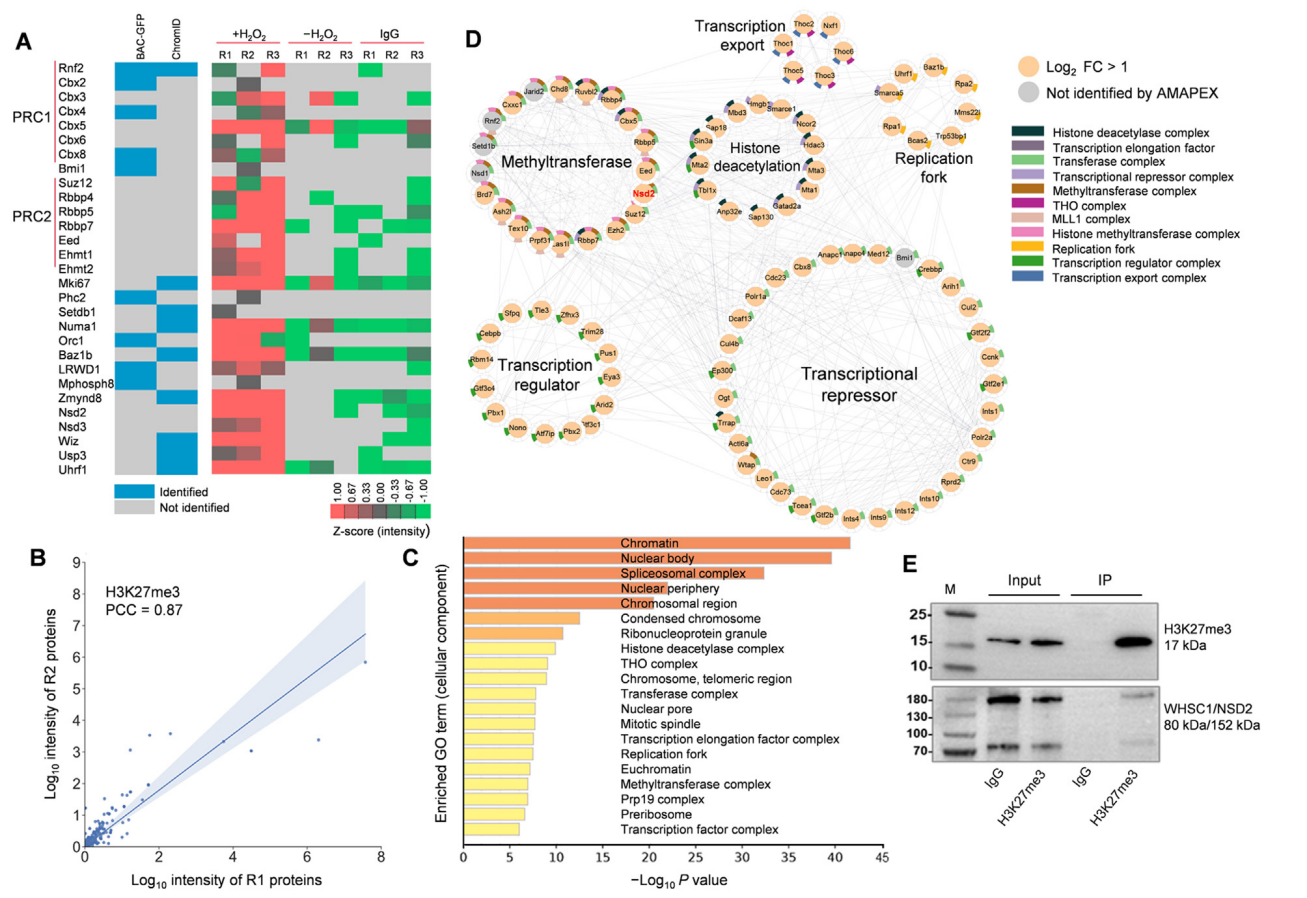
**The proximal proteome of H3K27me3 identified by AMAPEX** **A.** H3K27me3-proximal proteins identified by AMAPEX. Heatmap shows the log-transformed and mean-centered intensity of the proximal proteins derived from Maxquant processes of the raw protein MS data obtained by adding H_2_O_2_ (+H_2_O_2_). The experiments using IgG isotype (IgG) or without H_2_O_2_ (−H_2_O_2_) were included as controls. Experiments were performed with three biological replicates (R1, R2, and R3). Data are shown as Z scores. Blocks in blue represent the enrichment of proteins identified by ChromID and/or BAC-GFP in previous publications. **B.** The reproducibility of two biological replicates of +H_2_O_2_ experiments in the identification of H3K27me3-proximal proteins by calculating PCC. **C.** The top 20 enriched GO cellular component terms of H3K27me3-proximal proteins. Bar plots represent the −Log_10_*P* values of enriched terms. **D.** Network analysis of selected GO cellular component terms for H3K27me3-proximal proteome. GO cellular component terms with *P* < 1 × 10^−3^ for enrichment and related to histone modifications were selected for visualization here, involving 97 proteins out of the 494 proteins identified in the H3K27me3-proximal proteome. Individual proteins are shown as nodes, and interactions are shown as edges. The interactions were retrieved from the STRING database with interaction score > 0.4. Significantly enriched proteins (Log_2_ FC > 1) identified by AMAPEX in at least two out of three replicates are shown as orange nodes. Proteins detected by ChromID and/or BAC-GFP, but not reproducibly by AMAPEX, are shown as gray nodes. The protein selected for further analysis is highlighted in red. **E.** Mono-nucleosomes were purified from MEF cells (negative control) followed by IP. Proteins from input and IP samples were analyzed by Western blotting using the indicated antibodies. IgG represents a control antibody used for IP. AMAPEX, antibody-mediated pA-APEX2 labeling; PCC, Pearson correlation coefficient; GO, Gene Ontology; FC, fold change; WHSC1, Wolf-Hirschhorn syndrome candidate 1; NSD2, nuclear receptor binding SET domain protein 2.

**Supplementary Figure S2:**
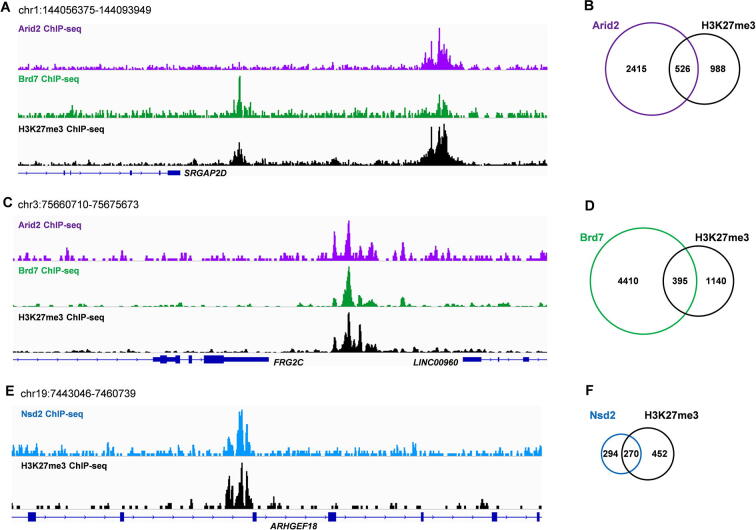
**The interactions between H3K27me3 and Arid2, Brd7, or Nsd2 are verified by ChIP-seq** Representative co-localization of ChIP-seq peaks between H3K27me3 and Arid2 (**A**), Brd7 (**C**) or Nsd2 (**E**). Venn diagrams show the number of H3K27me3 peaks overlapping with Arid2 (**B**), Brd7 (**D**) or Nsd2 (**F**) peaks. The ChIP-seq datasets for Arid2, Brd7, and H3K27me3 are from MCF-7 cells. The ChIP-seq dataset for Nsd2 and H3K27me3 are from K-562 cells. All of the bigwig files and peak files were downloaded from https://chip-atlas.org/.

To test whether pA-APEX2 could be used to identify proteins associated with histone modifications *in situ*, the biotinylated proteins were enriched with streptavidin beads and analyzed using quantitative liquid chromatography–tandem mass spectrometry (LC–MS/MS) (Figure 1B). Samples that were incubated with IgG or without H_2_O_2_ were included as negative controls. Compared to ChromID [14] and BAC-GFP [15], we reproducibly recovered most of the PRC1 and PRC2 subunits using quantitative LC–MS/MS (Figure 2A and B). In addition to the known H3K27me3-associated proteins, we identified a number of candidate proteins that have not been shown to associate with H3K27me3 (Table S1). Notably, Gene Ontology (GO) term and network analyses indicated that these proteins were enriched in the cellular components that are known to be associated with H3K27me3. These include transcription repressor complex [20], histone methyltransferase complex [20], and DNA replication fork [21], [22] (Figure 2C and D; Table S2). To validate this result, we performed mono-nucleosome H3K27me3 immunoprecipitation and found that one of the candidates, the H3K36 methyltransferase NSD2, could bind to H3K27me3-modified nucleosomes (Figure 2E). We further analyzed the published ChIP-seq datasets of three candidates: NSD2 and two polybromo-associated BAF (PBAF) subunits (Brd7 and Arid2), to assess their genome-wide enrichment [23] (Figure S2). We found that PBAF subunits Brd7 and Arid2 co-localized with H3K27me3 in MCF-7 cells globally (Figure S2A–D), indicating that PBAF may recognize H3K27me3 to remodel suppressed chromatin [24]. The ChIP-seq results also showed that around half of NSD2 peaks in K-562 cells overlapped with H3K27me3 peaks (Figure S2E and F), supporting our observation that NSD2 is proximal to H3K27me3. Together, these results demonstrate that AMAPEX could be used to identify the proximal proteins of modified histones.

### Comparison of APEX2- and HRP-mediated biotinylation by antibody recognition

**Figure 3:**
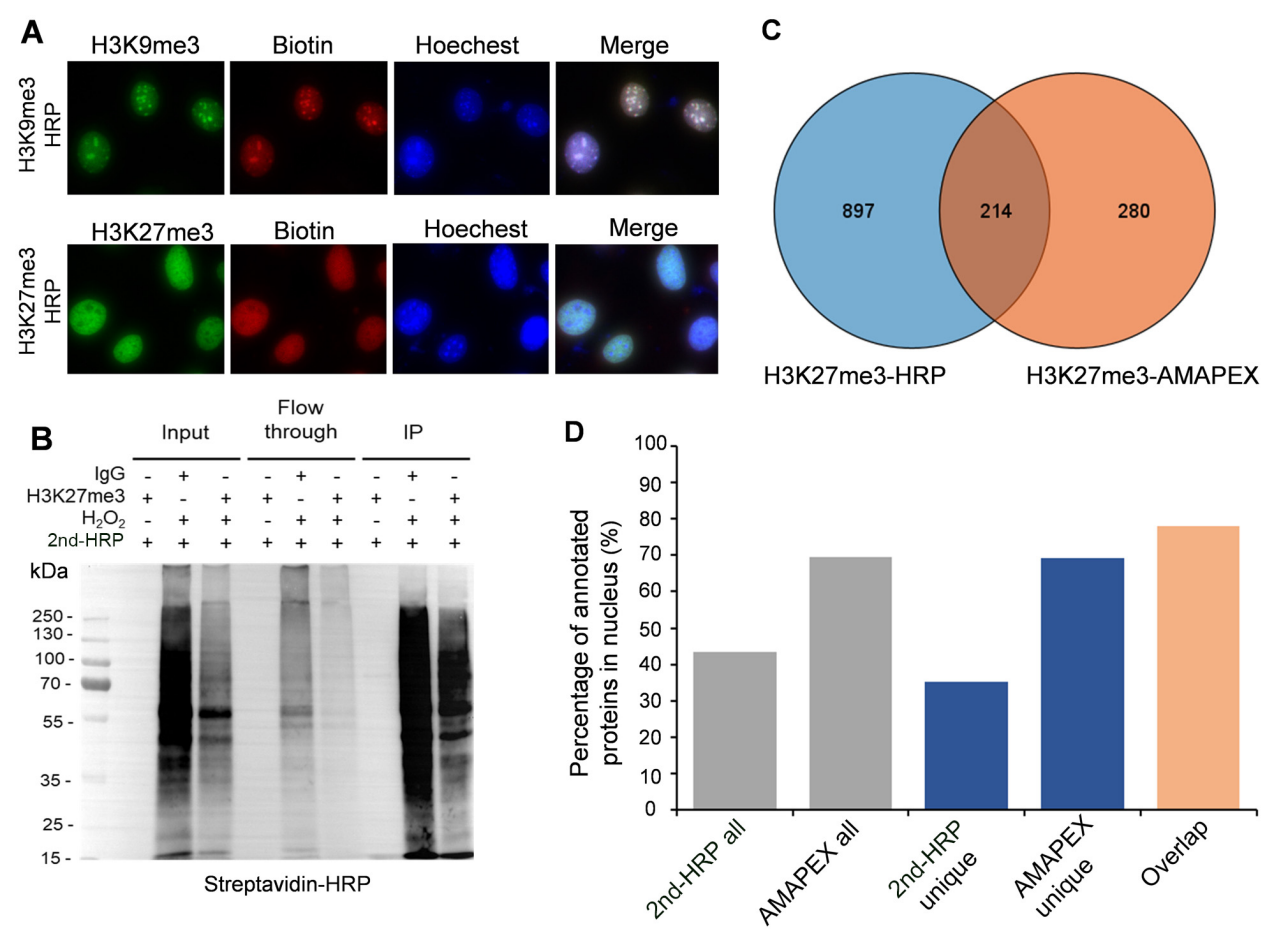
**Comparison of APEX2- and HRP-mediated biotinylation by antibody recognition** **A.** Fluorescence imaging of HRP-mediated biotinylation labeling in MEF cells. H3K9me3 and H3K27me3 were visualized by immunofluorescence staining (green). Biotinylation was induced by adding BP and H_2_O_2_ and visualized by staining with streptavidin-Cy3 (red). Nuclei were counterstained with Hoechest33342. **B.** Secondary HRP (2nd-HRP)-mediated protein labeling in whole-cell lysates. MEF cells were fixed with 4% formaldehyde, and incubated with H3K27me3 antibody and 2nd-HRP. Biotinylation was induced by adding BP and H_2_O_2_ and analyzed by Western blotting as indicated. **C.** Venn diagram of H3K27me3-proximal proteins identified by 2nd-HRP and AMAPEX. **D.** The percentages of H3K27me3-proximal proteins localized in the nucleus detected by HRP and AMAPEX methods. Percentages of proteins annotated as nuclear out of all proteins identified by each method (all), unique proteins only identified by 2nd-HRP or AMAPEX (unique), and common proteins identified by both methods are plotted. The nuclear location information was annotated according to the UniProt Database (https://www.uniprot.org/). HRP, horse radish peroxidase.

Horse radish peroxidase (HRP) has been used to label proximal proteins guided by antibodies in fixed cells and tissues [25]. We then asked whether APEX2 has better performance than HRP in terms of biotinylation by antibody recognition. Immunofluorescence and Western blotting assays showed that both APEX2 and HRP could robustly label nearby proteins of histone marks H3K27me3 and H3K9me3 (Figure 3A and B, Figure 1C); however, APEX2 showed a slightly better signal-to-noise ratio by immunofluorescence analysis.

We next analyzed the H3K27me3-proximal proteome by HRP-mediated biotinylation. The biotinylated proteins were enriched by streptavidin beads and identified using quantitative LC–MS/MS. Overall, more proteins were identified by HRP than APEX2. Interestingly, the identified proteins are highly overlapping, and among the proteins identified by H3K27me3-HRP, 214 could also be recovered by H3K27me3-AMAPEX (Figure 3C; Table S3). We then examined the subcellular localization of the proteins identified by H3K27me3-HRP and H3K27me3-AMAPEX, separately. The results showed that 69.4% of the proteins identified by H3K27me3-AMAPEX were localized in the nucleus, but only 33.31% of the H3K27me3-HRP identified proteins were localized in the nucleus (Figure 3D; Table S3). These results indicate that even though with similar efficiency, APMEX-mediated biotin labeling shows much lower background level compared to HRP-meditated labeling.

### Identification of histone modification proximal proteins via AMAPEX

**Figure 4:**
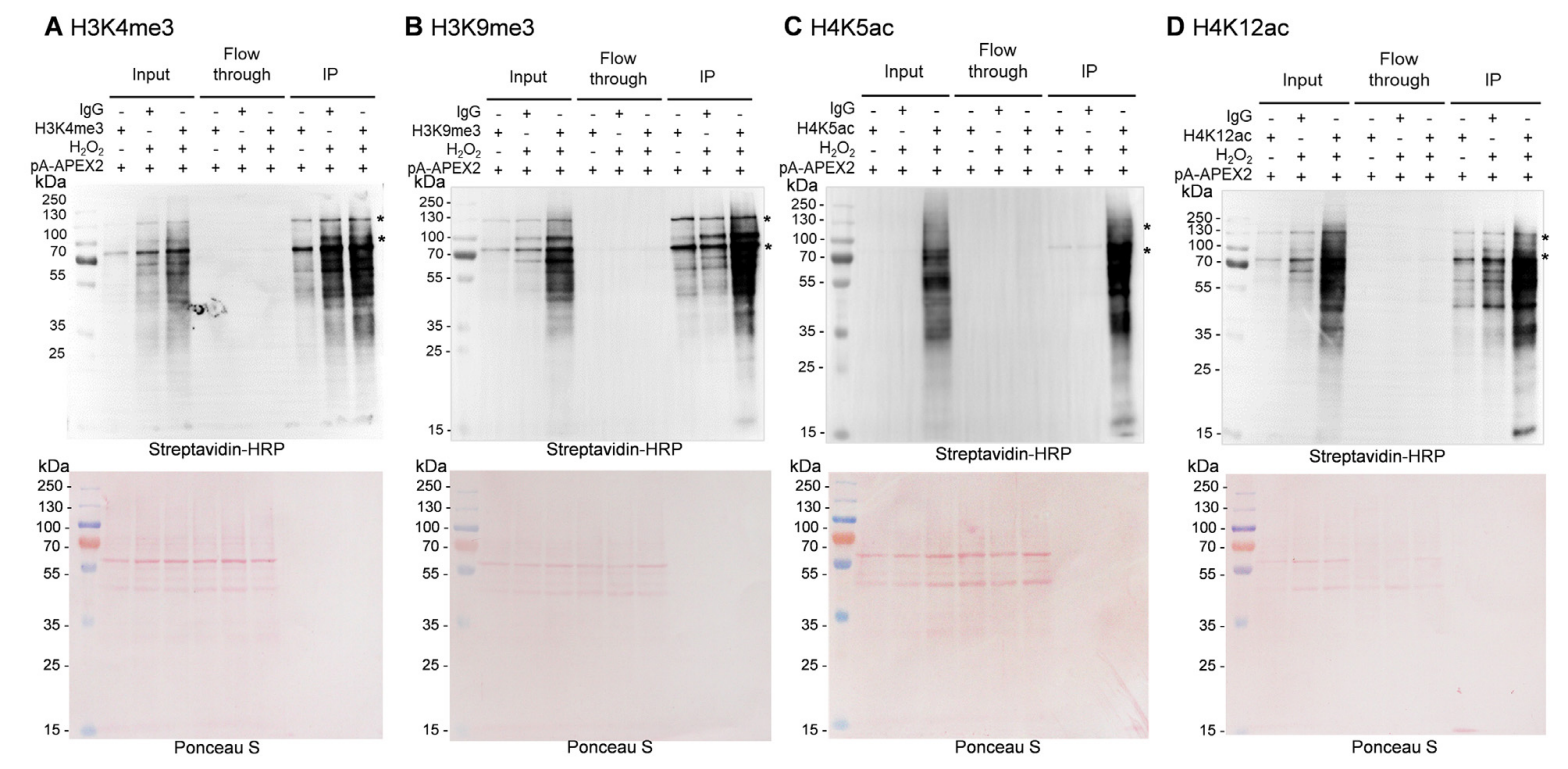
**Antibody-mediated specific biotin labeling by pA-APEX2** MEF cells were briefly crosslinked with 0.1% formaldehyde before the antibody-directed pA-APEX2 biotinylation reaction. Whole-cell lysates were extracted, and biotinylated proteins were purified using streptavidin beads. Whole-cell lysates (input), flow through, and IP samples were analyzed by Western blotting. **A.** H3K4me3. **B.** H3K9me3. **C.** H4K5ac. **D.** H4K12ac. Asterisks denote endogenous biotinylated proteins. Lower panels show Ponceau S staining as loading controls.

**Figure 5:**
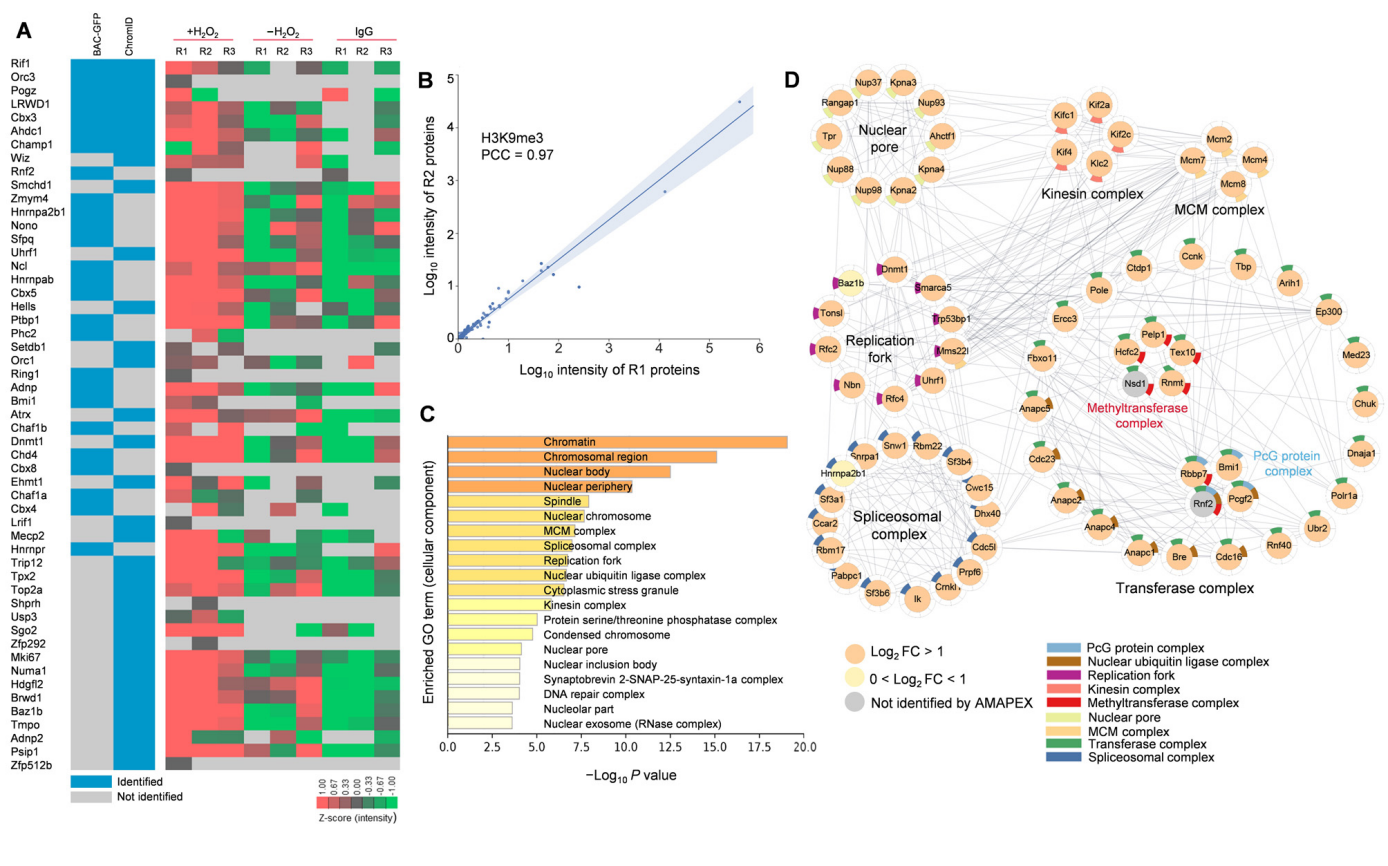
**The proximal proteome of H3K9me3 identified by AMAPEX** **A.** H3K9me3-proximal proteins identified by AMAPEX. Heatmap shows the log-transformed and mean-centered intensities of the proximal proteins derived from Maxquant processes of the raw protein MS data obtained by adding H_2_O_2_ (+H_2_O_2_). The experiments using IgG isotype or without H_2_O_2_ (−H_2_O_2_) were included as controls. Experiments were performed with three biological replicates (R1, R2, and R3). Data are shown as Z scores. Blocks in blue represent the enrichment of proteins identified by ChromID and/or BAC-GFP in previous publications. **B.** The reproducibility of two biological replicates of pA-APEX2 experiments in the identification of H3K9me3-proximal proteins by calculating PCC. **C.** The top 20 enriched GO cellular component terms for the H3K9me3-proximal proteins. Bar plots represent the −Log_10_*P* values of the enriched terms. **D.** Network analysis of selected GO cellular component terms for H3K9me3-proximal proteome. GO term selection and visualization methods were described in Figure 2D. In total 75 proteins out of the 486 proteins identified in the H3K9me3-proximal proteome were visualized here. Individual proteins are shown as nodes, and interactions are shown as edges. Significantly enriched proteins (Log_2_ FC > 1) identified by AMAPEX in at least two out of three replicates are shown as orange nodes. Proteins with 0 < Log_2_ FC < 1 and detected in ChromID and/or BAC-GFP are shown as light yellow nodes; proteins detected by ChromID and/or BAC-GFP, but not reproducibly by AMAPEX, are shown as gray nodes. The two pathways in the largest cluster are color-coded for differentiation.

We next generalized our method to map the interactomes of major histone modifications including H3K4me3, H3K9me3, H4K5ac, and H4K12ac. Western blotting demonstrated successful labeling and enrichment of the proximal proteins of these histone marks (Figure 4). We performed LC–MS/MS to identify the proteomes of these modifications. We first analyzed the proteins enriched by H3K9me3 labeling and recovered several known H3K9me3-binding proteins, including the reader proteins Lrwd1 [26], [27], Cbx5 (HP1α), and Cbx3 (HP1γ) [28], the H3K9 methyltransferases Ehmt1 and Setdb1, the DNA replication-dependent nucleosome assembly chaperones Chaf1a and Chaf1b, and the DNA methylation maintenance proteins Mecp2, Uhrf1, and DNMT1 (Figure 5A; Table S1). We also identified H3K9me3-binding proteins that were enriched in relevant functional complexes, such as the DNA replication fork, methyltransferase complex, and PcG protein complex [29] (Figure 5C and D). Therefore, this method could help us understand the mechanisms underlying diverse biological processes including epigenetic inheritance.

**Figure 6:**
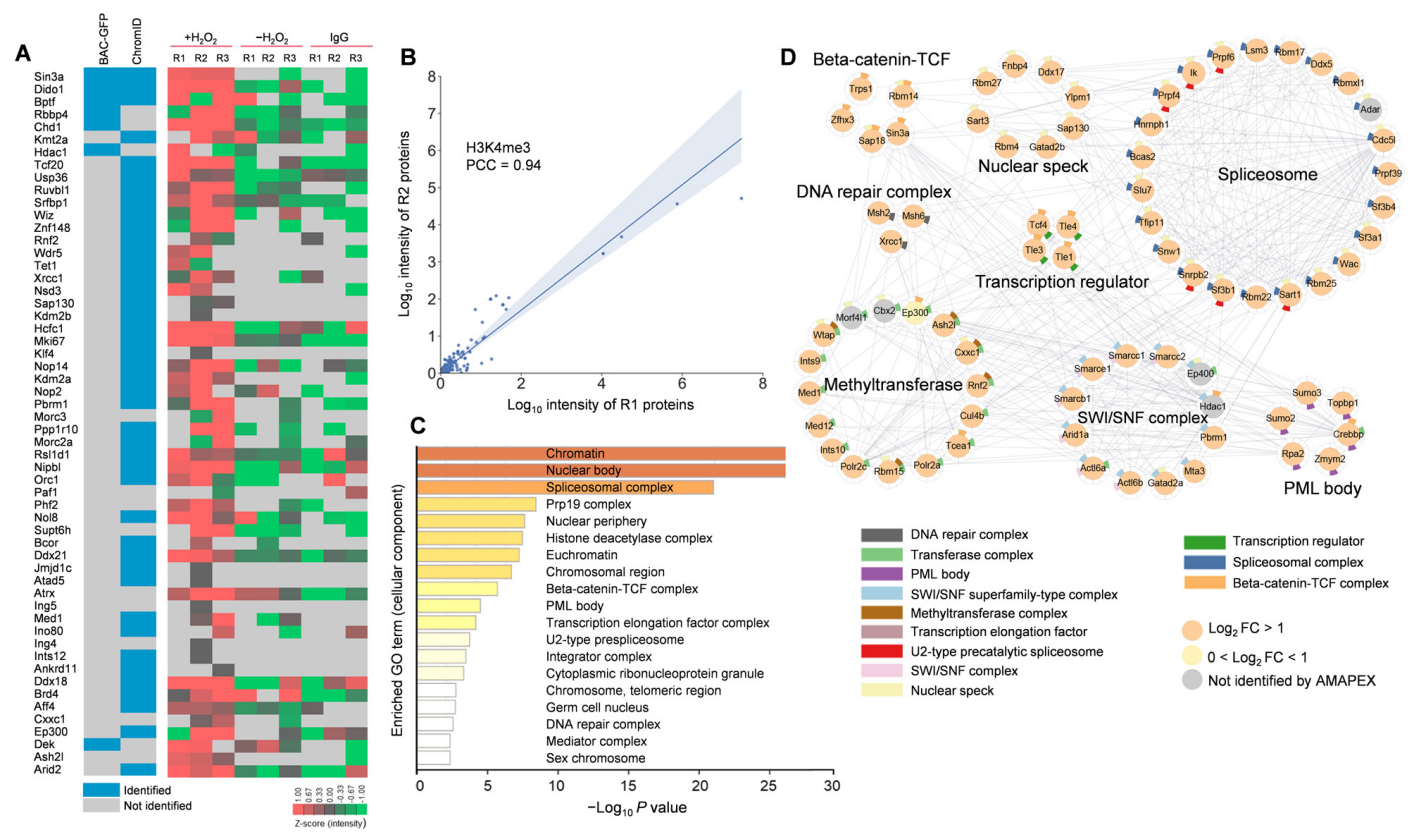
**The proximal proteome of H3K4me3 identified by AMAPEX** **A.** H3K4me3-proximal proteins identified by AMAPEX. Heatmap shows the log-transformed and mean-centered intensities of the proximal proteins derived from Maxquant processes of the raw protein MS data obtained by adding H_2_O_2_ (+H_2_O_2_). The experiments using IgG isotype or without H_2_O_2_ (−H_2_O_2_) were included as controls. Experiments were performed with three biological replicates (R1, R2, and R3). Blocks in blue represent the enrichment of proteins identified by ChromID and/or BAC-GFP in previous publications. **B.** The reproducibility of two biological replicates of pA-APEX2 experiments identifying H3K4me3-proximal proteins. PCC between two replicates was calculated. **C.** The top 20 enriched GO cellular component terms for the H3K4me3-proximal proteins. Bar plots represent the −Log_10_*P* values of the enriched terms. **D.** Network analysis of selected GO cellular component terms for H3K4me3-proximal proteome. GO term selection and visualization methods were described in Figure 2D. In total 77 proteins out of the 193 proteins identified in the H3K4me3-proximal proteome were shown here. Significantly enriched proteins (Log_2_ FC > 1) identified by AMAPEX in at least two out of three replicates are shown as orange nodes. Proteins with 0 < Log_2_ FC < 1 and detected in ChromID and/or BAC-GFP are shown as light yellow nodes; proteins detected by ChromID and/or BAC-GFP, but not reproducibly by AMAPEX, are shown as gray nodes.

**Figure 7:**
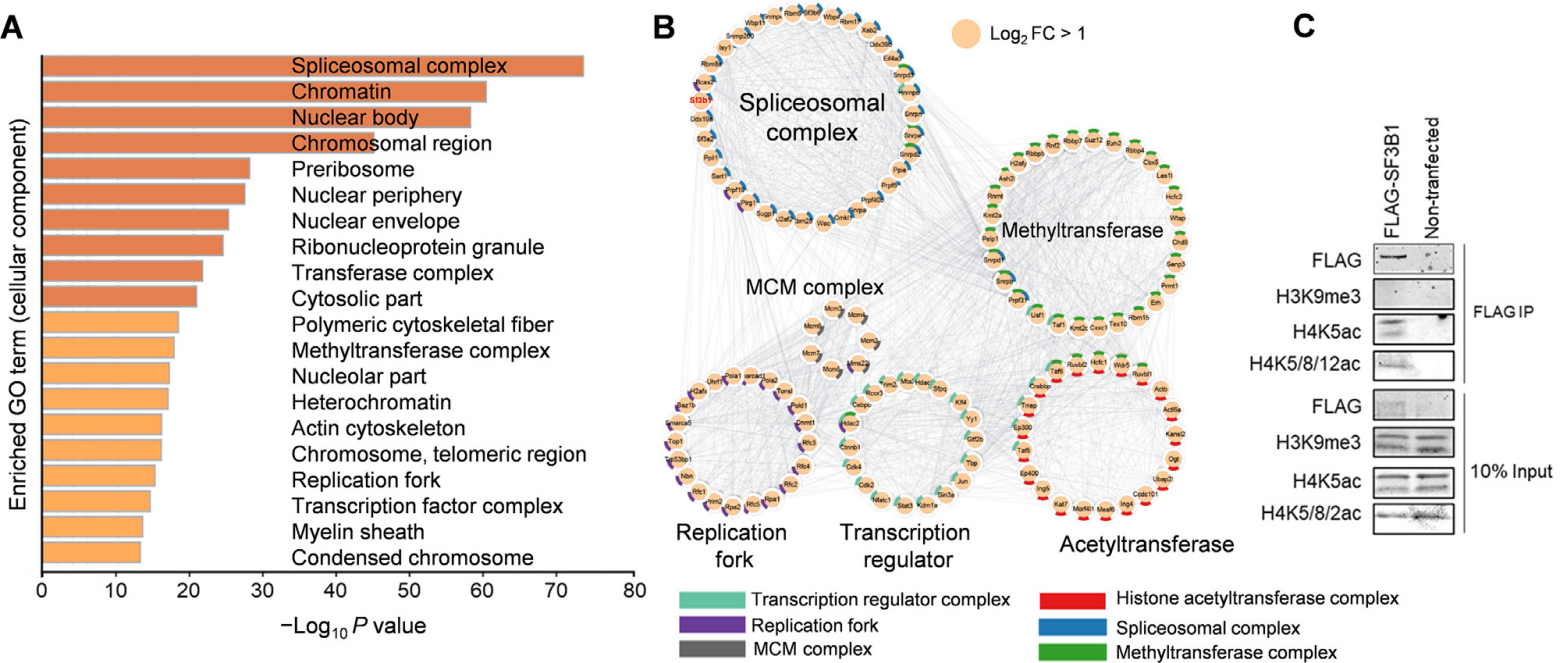
**The proximal proteome of H4K5ac identified by AMAPEX** **A.** The top 20 enriched GO cellular component terms for the H4K5ac-proximal proteins. Bar plots representing the −Log_10_*P* value of the enriched terms. **B.** Network analysis of selected GO cellular component terms for H4K5ac-proximal proteome. GO term selection and visualization methods were described in Figure 2D. In total 117 proteins out of the 1783 proteins identified in the H4K5ac-proximal proteome were shown here. Proteins detected by AMAPEX in at least two of three replicates with Log_2_ FC > 1 are shown as orange nodes. The protein selected for further analysis is highlighted in red. **C.** Mono-nucleosomes were purified from FLAG-SF3B1 expressing-HEK293T cells and non-transfected HEK293T cells (negative control) followed by IP. Proteins from input and IP samples were analyzed by Western blotting using the indicated antibodies.

**Supplementary Figure S3:**
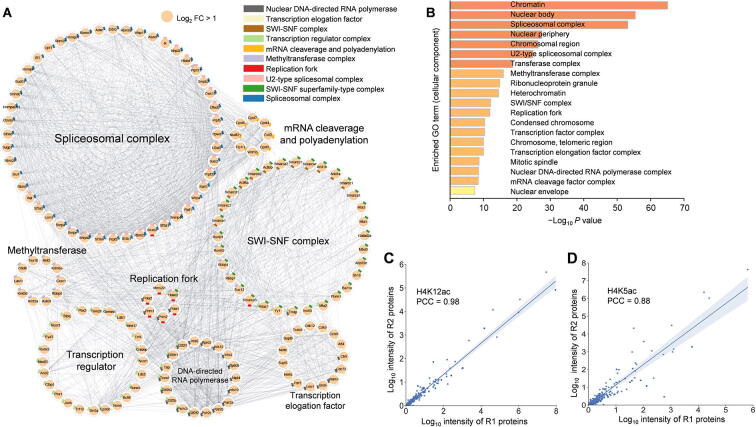
**The proximal proteome of H4K12ac identified by AMAPEX A.** Network analysis of selected GO cellular component terms for H4K12ac-proximal proteome. GO cellular component terms with *P* < 10^−3^ for enrichment and related to histone modifications were selected for visualization here, involving 161 proteins out of the 1019 proteins identified in the H4K12ac-proximal proteome. Individual proteins are shown as nodes, and interactions are shown as edges. The interactions were retrieved from the STRING database with interaction score > 0.4. Experiments were performed with two biological replicates and proteins detected by AMAPEX in both replicates with Log_2_ FC > 1 are shown as orange nodes. **B.** The top 20 cellular component GO terms for the H4K5ac-proximal proteins. Bar plots represent the −Log_10_*P* values of the enriched terms. **C.** The reproducibility of two biological replicates of pA-APEX2 experiments identifying H4K12ac-proximal proteins. **D.** The reproducibility of two biological replicates of pA-APEX2 experiments identifying H4K5ac-proximal proteins. PCC between two replicates was calculated.

In addition, we also recovered the H3K4 methyltransferase Kmt2a and COMPASS-related proteins including Wdr5 and Cxxc1 [30] in the H3K4me3 interactome (Figure 6A and B; Table S1). As expected, H3K4me3-proximal proteins were mostly enriched in the pathways related to active transcription including RNA splicing and euchromatin (Figure 6C and D). We next focused on proteins that interact with H4K5ac, the histone modification that marks newly synthesized histones [31]. The H4K5ac interactome was enriched with minichromosome maintenance (MCM) complex [32] and DNA replication fork proteins (Figure 7A and B; Table S2), indicating that newly synthesized H3/H4 complexes are deposited on DNA in a replication-dependent manner. However, we did not observe association between H4K12ac-labeled proteins and DNA replication (Figure S3A–C), suggesting that the H4K12ac modification may not be recognized by new histone H3/H4 deposition machinery. The proteins identified by H4K5ac-AMAPEX and H4K12ac-AMAPEX were enriched in RNA spliceosomes. Then, we validated the interaction between H4K5ac and a major RNA spliceosome protein SF3B1 by mono-nucleosome immunoprecipitation (Figure 7C). These results indicate that H4K5ac may be involved in RNA splicing.

**Supplementary Figure S4:**
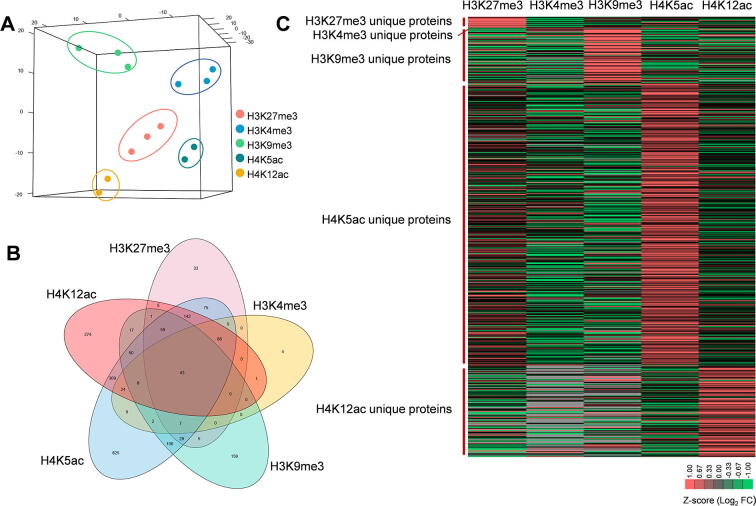
**The specificity of the proteome identified by H3K27me3, H3K4me3, H3K9me3, H4K5ac, and H4K12ac AMAPEX A.** PCA of identified proximal proteins by H3K27me3, H3K4me3, H3K9me3, H4K5ac, and H4K12ac AMAPEX. The Log_2_ FC values of proteins identified by the AMAPEX experiment versus −H_2_O_2_ were used to run the PCA. Each dot represents one biological replicate, and the first three PCs were displayed. The PCA was implemented by packages ggplot2 (version 3.3.3) and rgl (version 0.105.13) in R (version 4.0.2). **B.** Venn plot showing overlaps of AMAPEX-identified proximal proteins among five differential histone modifications, including H3K27me3, H3K4me3, H3K9me3, H4K5ac, and H4K12ac. **C.** The proximal proteins uniquely identified in H3K27me3 (33), H3K4me3 (4), H3K9me3 (159), H4K5ac (825), and H4K12ac (274) and their normalized intensities in other types of histone modifications. The Log_2_ FC values of the AMAPEX experiment versus −H_2_O_2_ identified proteins were adopted and adjusted by log transformation and mean-centered in Cluster (version 3.0), and visualized by TreeView (version 1.2.0).

Globally, we compared the proximal proteomics landscape of all the histone modifications we tested using unsupervised principal component analysis (PCA), which revealed the separation of different histone modifications (Figure S4A). Except for the H3K4me3-AMAPEX, which only captured 4 unique ones (Figure S4B), AMAPEX of other four histone marks identified a considerable number of unique proximal proteins (Figure S4B). We observed 43 commonly picked proteins. Among them, some are general H3-binding proteins, and others may be false positives that could be seen using any MS-based strategies. Together, AMAPEX can identify unique proximal proteins with specific antibodies (Figure S4C).

### Identification of histone modification proximal proteins via AMAPEX under native conditions

**Figure 8:**
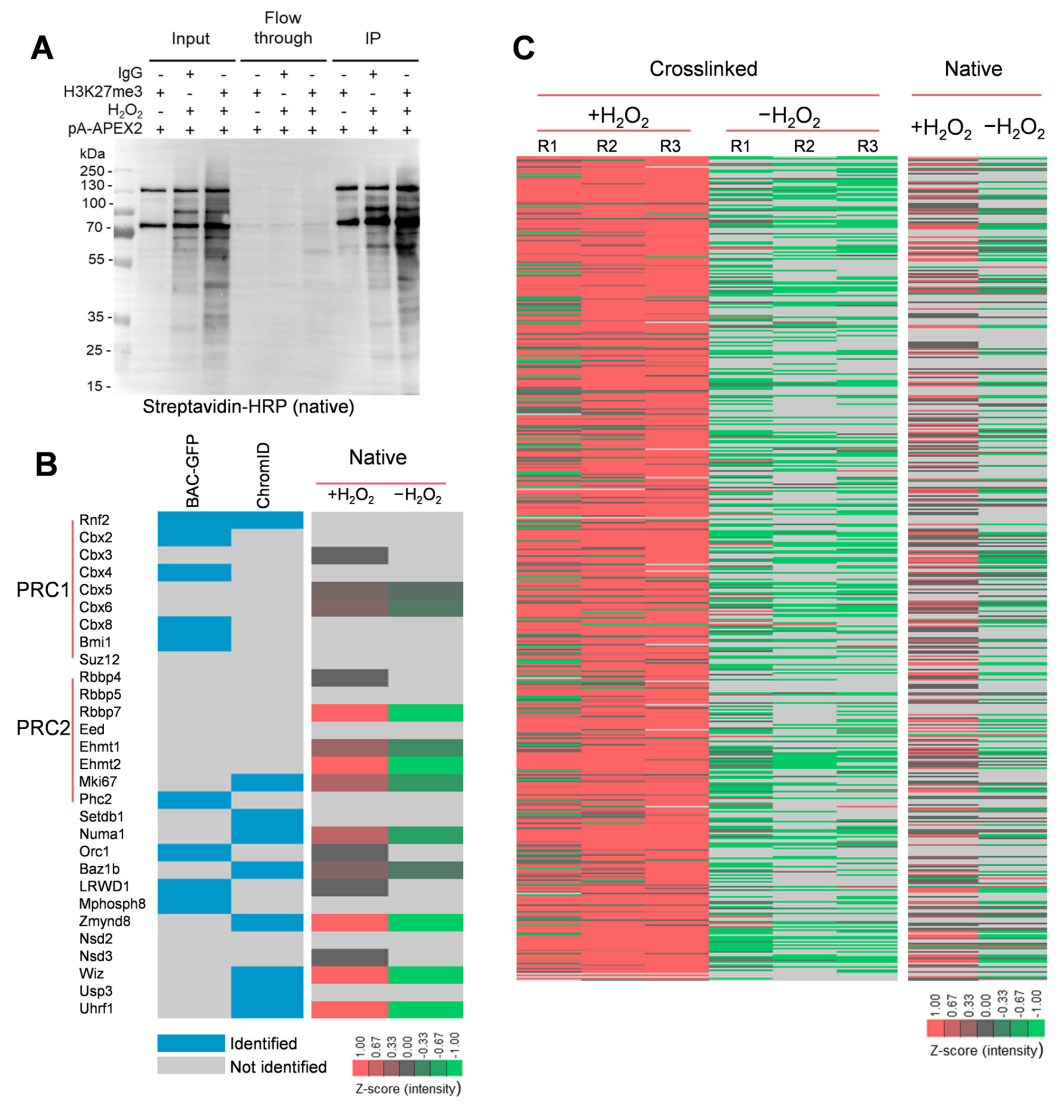
**Identification of histone modification-proximal proteins via AMAPEX under native conditions** **A.** pA-APEX2-mediated protein labeling in whole-cell lysates under native conditions. Whole-cell lysates were extracted, and biotinylated proteins were purified using streptavidin beads. Whole-cell lysates (input), flow through, and IP samples were analyzed by Western blotting. **B.** Identified H3K27me3-proximal proteins by native AMAPEX. Blocks in blue represent the enrichment of proteins identified by ChromID and/or BAC-GFP in previous publications. Heatmap on the right represents the log-transformed and mean-centered intensities of the proximal proteins derived from Maxquant processes of the raw protein MS data obtained from +H_2_O_2_ and −H_2_O_2_ experiments, respectively. **C.** Heatmap showing the AMAPEX-identified H3K27me3-proximal proteomes under crosslinked (on the left) and native (on the right) conditions. Experiments were performed with three biological replicates (R1, R2, and R3) under the crosslinked condition.

Formaldehyde crosslinking may cause artifacts [33], [34]; therefore, we asked whether AMAPEX could be applied under native conditions. We performed AMAPEX under native conditions to label the nearby proteins of H3K27me3. The results showed effective biotinylation by H3K27me3 antibody but not IgG control or without H_2_O_2_, suggesting that AMAPEX could also label nearby proteins in native protein samples (Figure 8A). To further test if AMAPEX could identify proteins associated with histone modifications *in situ* under native conditions, the biotinylated proteins were enriched with streptavidin beads and analyzed by quantitative LC–MS/MS (Figure 8B; Table S3); samples incubated without H_2_O_2_ were included as negative controls. Compared to ChromID and BAC-GFP, we could still robustly identify known H3K27me3 proximal proteins under native conditions using quantitative LC–MS/MS (Figure 8C; Table S3). We also found that native H3K27me3-AMAPEX could identify most of the proteins showed in the crosslinked H3K27me3-AMAPEX (Figure 8C). These results demonstrated that AMAPEX could also be applied to explore histone modifications under native conditions.

## Discussion

To identify the proximal proteins of histone modifications, we developed a method named AMAPEX to label the nearby proteins via antibody-mediated biotinylation. We identified proximal complexes of histone modifications without the expression of exogenous fusion proteins. Both previously reported and novel interactors of these histone modifications were identified by our method. We successfully validated some of the newly identified proximal proteins. For example, the histone H3K36 di-methyltransferase NSD2 could bind to H3K27me3 mono-nucleosomes. H3K36me2 is a negative regulator of H3K27me3 [35] and has been demonstrated to be a boundary barrier of PRC-mediated H3K27me3 spreading [36]. On the other hand, H3K27me3 also inhibits H3K36me2. Therefore, it is possible that the H3K27me3 nucleosome might inhibit the activity of NSD2 to suppress H3K36me2 spreading. This result provides new insights into the mechanisms underlying epigenetic spreading. We also showed that the RNA spliceosome protein SF3B1 binds to H4K5ac-modified nucleosomes. Although H4K5ac is considered as a PTM mark for newly synthesized histones, our results implicate its potential novel role in RNA splicing. Future biochemical and functional analyses would provide more insights into the functions of H4K5ac.

Most of the approaches used in chromatin biology studies, including ChIP, ChIP-MS [19], Hi-C [37], and HiChIP [38], require formaldehyde crosslinking. Nevertheless, a variety of proteins including transcription factors bind to their target temporally in a hit-and-run manner [39], therefore crosslinking would allow us to capture these transient interactions by AMAPEX. Importantly, since our approach does not require the expression of exogenous proteins, AMAPEX can be potentially applied to the formalin-fixed paraffin-embedded (FFPE) samples. However, in some cases crosslinking might cause undesired effects as well. For instance, the nuclear proteins/loci cannot be crosslinked at equal efficiency [34]. In addition, crosslinking can also trigger DNA damage response [33], and in some cases mask the epitopes of antibodies, thereby affecting antibody/antigen binding. To overcome the drawbacks of crosslinking-AMAPEX, we also tested AMAPEX under native conditions, and successfully identified the known proteins that form complexes with H3K27me3 (PRC1 and PRC2). Native AMAPEX could also identify proteins that were not detected by crosslinking-APAPEX, possibly because under crosslinking, epitopes of some antibodies are masked. These results suggest that AMAPEX is also feasible under native conditions.

AMAPEX identifies unique proximal proteins for different histone modifications, as well as common proteins recovered by multiple histone marks. Some of the proteins captured by multiple histone modifications might represent their real biological functions. For example, proteins bind to the bivalent promoter regions, which are marked with both H3K4me3 and H3K27me3. However, we did observe some false positives, which were commonly picked up by all different histone marks. Therefore, careful validations are necessary to be integrated into a complete AMAPEX workflow before one can claim the discovery of a new histone modification binder, as with many other antibody- and MS-based methods. Even so, we have shown that AMAPEX can successfully provide clues to novel proximal proteins with the specific antibodies.

In summary, our novel method features two major advances. First, we demonstrate that AMAPEX is an efficient proximity labeling tool that is not dependent on the expression of exogenous fusion proteins, making it possible to profile proximal proteomes in primary cell lines or tissues. Second, AMAPEX can also robustly map proximal proteins of PTM proteins, under both fixed and native conditions. Utilizing this method, we successfully profiled proximal proteins of five different histone marks and uncovered several very promising protein–histone mark pairs. Future functional exploration of our findings would shed new lights on the chromatin biology studies.

## Materials and methods

### Antibodies

Detailed information on antibodies used in this study can be found in Table S4.

### Plasmid construction

The fragment of *APEX2* was amplified from the GFP-APEX2-NIK3x plasmid (a gift from Alice Ting; Catalog No. 129274, Addgene) by PCR, and then inserted into the *Nde*I and *Spe*I sites of the 3×FLAG-pA-Tn5-Fl plasmid (Catalog No. 124601, Addgene) to generate the 3×FLAG-pA-APEX2 plasmid.

### Purification of the 3×FLAG-pA-APEX2 protein

Protein purification was performed as previously described [40]. The 3×FLAG-pA-APEX2 plasmid was transformed into C3013 cells and incubated overnight at 37 °C. A single colony was selected and inoculated into 3 ml LB medium, and growth was continued at 37 °C for 4 h. This culture was used to start a culture in 400 ml LB medium containing 100 µg/ml carbenicillin and incubated on a shaker until it reached OD ∼ 0.6; the culture was then chilled on ice for 30 min. Fresh IPTG (Catalog No. I6758, Sigma, Chicago, IL) was added to a final concentration of 0.25 mM to induce protein expression, and the culture was incubated at 18 °C on a shaker overnight. The culture was collected by centrifugation at 6000 *g* and 4 °C for 30 min. The pellet was stored at −80 °C until processing. The protein purification steps were as follows. Briefly, a frozen pellet was resuspended in 40 ml chilled HEGX Buffer (20 mM HEPES-KOH pH 7.2, 1 M NaCl, 1 mM EDTA, 10% glycerol, 0.2% Triton X-100) supplemented with 1× Roche Complete EDTA-free protease inhibitor tablets (Catalog No. M9260G, Invitrogen, Carlsbad, CA) and kept on ice for 15 min. The lysate was sonicated for 15 min (300 W, 3 s on, 5 s off) on ice. The sonicated lysate was centrifuged at 16,000 *g* at 4 °C for 30 min, and the soluble fraction was moved to fresh 50-ml tubes. A 4-ml aliquot of chitin resin (Catalog No. S6651S, NEB, Ipswich, MA) was packed into each of two disposable columns (Catalog No. 7321010, Bio-Rad, Hercules, CA). Columns were washed with 20 ml HEGX Buffer. The supernatant was added to the chitin resin slowly and then incubated on a rotator at 4 °C for 1 h. The unbound soluble fraction was drained, and the columns were washed twice with 20 ml HEGX buffer. The chitin slurry was transferred to a 15-ml tube and resuspended in elution buffer [6 ml HEGX buffer supplemented with 100 mM DTT (Catalog No. D0632, Roche, Switzerland)]. The tube was placed on a nutator at 4 °C for 60 h. The eluate was collected and dialyzed twice in 1 l dialysis buffer (100 mM HEPES-KOH pH 7.2, 0.2 M NaCl, 0.2 mM EDTA, 2 mM DTT, 0.2% Triton X-100, 20% glycerol). The dialyzed protein solution was concentrated using Amicon Ultra-4 Centrifugal Filter Units 30 K (Catalog No. UFC803024, Millipore, MA), and sterile glycerol was added to make a final 50% glycerol stock of the purified protein. The purified protein was aliquoted and stored at −20 °C. The pA-APEX2 purification was analyzed by SDS-PAGE. The concentration of pA-APEX2 was determined using BSA standards.

### Mammalian cell culture

MEF cells were cultured in DMEM/high glucose supplemented with 10% fetal bovine serum, 100 U/ml penicillin, and 100 mg/ml streptomycin at 37 °C under 5% CO_2_. Mycoplasma testing was performed before experiments.

### Immunofluorescence staining and fluorescence microscopy

MEF cells were fixed with 4% paraformaldehyde in PBS at room temperature for 15 min. Cells were then washed with PBS three times and blocked for 1 h with 3% BSA in 0.1% PBST (blocking buffer) at room temperature. Cells were incubated with primary antibodies [rabbit anti-H3K9me3 antibody (1:100; Catalog No. ab8898, Abcam, Cambridge, UK); rabbit anti-H3K27me3 antibody (1:100; Catalog No. 9733S, Cell Signaling Technology, Poston, MA)] in blocking buffer for 1 h at room temperature. After washing three times with PBS, cells were incubated with pA-APEX2 (generated in this study, 4 μg/μl, 1:400) in blocking buffer for 1 h and then washed three times with PBST. Next, cells were incubated with 500 μM BP in PBS at room temperature for 30 min. H_2_O_2_ then was added to each well to a final concentration of 1 mM, and the plate was gently agitated for 1 min. The reaction was quenched with an equal volume of 2× quench buffer (10 mM Trolox, 20 mM sodium ascorbate, and 20 mM sodium azide in PBS). Samples incubated with IgG and without BP (no-BP) were included as negative controls. After washing three times with PBST, cells were incubated with secondary antibodies [Alexa Fluor 488 (1:200; Catalog No. A32790, Invitrogen); streptavidin-Cy3 (1:300; Catalog No. S6402, Sigma)] in blocking buffer for 1 h at room temperature. Cells were washed and incubated with Hoechst for 10 min at room temperature, washed three times with PBS, and imaged.

### AMAPEX *in vitro*

Total protein of MEF cells was extracted with RIPA lysis buffer (50 mM Tris-HCl pH 7.5, 150 mM NaCl, 1.5 mM MgCl_2_, 1 mM EGTA, 0.1% SDS, 1% NP-40, 0.4% sodium deoxycholate, 1 mM DTT, 1 mM PMSF, and 1× Roche Complete EDTA-free protease inhibitor tablets) for 15 min at 4 °C. Cell extracts were sonicated (100 W, 3 s on, 3 s off) for 3 min. Cell extracts were clarified by centrifugation, and the amount of protein in each supernatant was measured. Afterward, 20 µg total protein was incubated with 10 μM pA-APEX2 and 0.5 mM BP in PBS for 1 min. The reaction was triggered by mixing with 1 mM H_2_O_2_ and stopped with quench buffer. Without H_2_O_2_, no-BP, or pA-APEX2 samples were included as negative controls.

### AMPEX in MEF cells

The labeling was adapted and modified from the CUT&Tag method [40]. A detailed, step-by-step AMAPEX protocol can be found in File S1. In total, 1 × 10^7^ cells were washed with 10 ml PBS, pooled into a 50-ml tube, and centrifuged at 250 *g* for 5 min. The cell pellet was resuspended in 1 ml PBS, crosslinked with freshly prepared formaldehyde at a final concentration of 0.1% at room temperature for 15 min, and quenched with 1/10 volume of 1.25 M glycine. The tube was inverted several times, shaken gently for 5 min, and centrifuged at 500 *g* for 5 min. The pellet was then washed once with 10 ml PBS and centrifuged at 500 *g* for 5 min. Supernatant was carefully aspirated, and the cell pellet was resuspended in 1 ml wash buffer (20 mM HEPES pH 7.5, 150 mM NaCl, 0.5 mM spermidine, 1× Protease Inhibitor EDTA-free tablet), transferred to a 1.5-ml tube, and centrifuged at 500 *g* for 5 min. The pellet was resuspended in 300 µl antibody buffer (4 µl 0.5 M EDTA, 3.3 µl 30% BSA, and 10 µl 5% digitonin in 1 ml wash buffer) supplemented with 2 µl primary antibody and incubated overnight at 4 °C. Then, the pellet was washed twice with 1 ml 0.01% digitonin wash buffer (20 µl 5% digitonin in 10 ml wash buffer) and centrifuged at 500 *g* for 5 min. The pellet was incubated with 300 µl of 500 µM BP in digitonin wash buffer for 30 min before incubation with 3 µl of 100 mM H_2_O_2_ in wash buffer for 1 min (at a final concentration of 1 mM H_2_O_2_). The reaction was quenched by adding 300 µl 2× quench buffer (20 mM sodium azide, 20 mM sodium ascorbate, 10 mM Trolox in wash buffer) [5]. Then, the pellet was washed twice with quench buffer. After carefully aspirating the supernatant, the cell pellet was flash frozen and stored at −80 °C until use.

### Streptavidin pull-down of biotinylated proteins and Western blotting analysis

pA-APEX2-labeled cell pellets were lyzed in RIPA lysis buffer (50 mM Tris-HCl pH 7.5, 150 mM NaCl, 1.5 mM MgCl_2_, 1 mM EGTA, 1% SDS, 1% NP-40, 0.4% sodium deoxycholate, 1 mM DTT, 1 mM PMSF, and 1× Roche Complete EDTA-free protease inhibitor tablets) for 15 min on ice. Cell extracts were sonicated (100 W, 3 s on, 3 s off) for 3 min and then boiled for 10 min at 100 °C. Cell extracts were clarified by centrifugation, and the amount of protein in each supernatant was measured. Afterward, 5% of the supernatant was saved as input for Western blotting analysis. SDS in the sample was diluted to 0.2% with 1× cold RIPA buffer (RIPA buffer without SDS). Streptavidin–Sepharose beads (Catalog No. 17-5113-01, GE Healthcare, Shanghai, China) were washed twice with 1× cold RIPA buffer (0.2% SDS), and 800 μg of each sample was separately incubated with 50 µl bead slurry with rotation for 4 h at 4 °C. Then 5% of the flow through was saved for Western blotting analysis. The beads were subsequently washed twice with 1 ml wash buffer (50 mM Tris-HCl pH 7.5, 1% SDS), twice with 1 ml RIPA wash buffer (50 mM Tris-HCl pH 7.5, 150 mM NaCl, 1.5 mM MgCl_2_, 1 mM EGTA, 0.2% SDS, 1% NP-40, 1 mM DTT), twice with 1 ml 8 M urea buffer, twice with 1 ml 30% acetonitrile (ACN), and twice with 1 ml 20 mM ammonium bicarbonate. Then, 5% of the beads were saved for Western blotting analysis, and the remaining beads were used for LC–MS/MS analysis. For Western blotting analysis, biotinylated proteins were eluted by boiling the beads in 10 µl 5× protein loading buffer and separated by 10% SDS-PAGE. The proteins were transferred to 0.22-μm PVDF membrane and stained with Ponceau S. The blots were then blocked in 1% BSA in TBST at room temperature for 1 h and stained with streptavidin-HRP (1:5000; Catalog No. A0303, Beyotime, Shanghai, China) in TBST for 1 h at room temperature. Blots were then washed with TBST buffer three times for 5 min, developed with Clarity Western ECL (Catalog No. 1705060, Bio-Rad) substrate, and imaged using a ChemiDoc MP Imaging System (Bio-Rad).

### On-bead digestion and LC–MS/MS

MS-based proteomic experiments were performed as previously described with minor modifications [41]. Briefly, after enrichment and washing, beads were resuspended in 200 µl on-bead digestion buffer (50 mM HEPES pH 8.0, 1 μM CaCl_2_, 2% ACN), and then 10 mM Tris(2-carboxyethyl) phosphine (TECP; Catalog No. 77720, ThermoFisher Scientific, Waltham, MA) and 40 mM chloroacetamide (CAA; Catalog No. 194921, Sigma) were added and incubated for 30 min at room temperature. The beads were washed with 1 ml on-bead digestion buffer. The beads were resuspended in 100 µl on-bead digestion buffer with endopeptidase (Catalog No. 125-05061, Wako Pure Chemical Industries, Osaka, Japan) and incubated at 37 °C for 3 h. Then, on-bead digestion buffer with 0.5 µg trypsin (Catalog No. V5280, Promega, Madison, WI) was added for digestion at 37 °C for 16 h.

The samples were desalted using StageTips before LC–MS/MS analysis. The StageTips were made of C18 material inserted in 200 μl pipette tips. To desalt the peptide samples, C18 material was washed once with 200 μl ACN, once with 200 μl StageTips buffer B [0.1% (v/v) formic acid (FA) in 50% (v/v) ACN/H_2_O], and twice with 100 μl StageTips buffer A [0.1% (v/v) FA in H_2_O]. Peptide samples were loaded on StageTips and washed twice with 100 μl StageTips buffer A. Finally, peptide samples were eluted with 100 μl StageTips buffer C [0.1% (v/v) FA in 40% (v/v) ACN/H_2_O] and 100 μl StageTips buffer B. The solutions were passed through the StageTips by centrifugation at 500 *g* for 5 min at room temperature. The elution fractions were collected, and the solution was evaporated from peptide samples in a SpeedVac at 45 ℃. Finally, 10 μl StageTips buffer A was added to the samples to perform LC–MS/MS analysis.

### LC–MS/MS analysis

All peptides were reconstituted in 0.1% (v/v) FA in H_2_O and separated on reversed-phase columns [trapping column: particle size = 3 μm, C18, length = 20 mm (P/N 164535, ThermoFisher Scientific); analytical column: particle size = 2 μm, C18, length = 150 mm (P/N 164534, ThermoFisher Scientific)] on an Ultimate 3000 RSLCnano system (ThermoFisher Scientific) coupled to Orbitrap Q-Exactive HF (ThermoFisher Scientific). Peptide separation was achieved using a 60-min gradient (buffer A: 0.1% FA in H_2_O; buffer B: 0.1% FA in 80% ACN/H_2_O) at a flow rate of 300 ml/min and analyzed by Orbitrap Q-Exactive HF in a data-dependent mode. The Orbitrap Q-Exactive HF mass spectrometer was operated in positive ion mode with ion transfer tube temperature set as 275 °C. The positive ion spray voltage was 2.1 kV. Full-scan MS spectra (*m/z* 350–2000) were acquired in the Orbitrap with a resolution of 60,000. Higher collisional dissociation (HCD) fragmentation was performed at normalized collision energy of 28%. The MS2 automatic gain control (AGC) target was set to 5E4 with a maximum injection time (MIT) of 50 ms, and dynamic exclusion was set to 30 s.

### MS data analysis

#### Protein identification and label-free protein quantification

Raw data were processed with MaxQuant (version 1.6.10.43) and its built-in Andromeda search engine for feature extraction, peptide identification, and protein inference. Mouse reference proteome from UniProt Database (UniProtKB/Swiss-Prot and UniProtKB/TrEMBL, version 2020_12) combined with manually annotated contaminants were applied to search the peptides and proteins. The false discovery rate (FDR) was set to 0.01, and a match-between-runs algorithm was enabled. After searching, the reverse hits, contaminants, and proteins only identified by one site were removed. Filtered results were exported and further visualized using the statistical computer language Python (version 3.8.3), the online gene annotation and analysis tool Metascape (version 2021_02), and the complex network visualizing platform Cytoscape (version 3.8.2).

#### Interacting protein detection of each specific histone mark

First, raw data were analyzed in MaxQuant using the basic principles as described above. Search results were filtered at FDR of 0.01 on precursor and protein group levels. The Pearson correlation coefficients (PCC) of all replicates were calculated using the function Series.corr() in Python library pandas (version 1.0.5). For each histone mark, the transformed protein intensities expressed as fold change (Log_2_ FC) in both the pA-APEX2 experiment compared to without H_2_O_2_ control experiment and the pA-APEX2 experiment compared to the IgG control experiment were calculated. For proteins that were not identified in the pA-APEX2 experiments but identified in the without H_2_O_2_ and/or IgG controls, the Log_2_ FC values were defined as −100. For proteins identified in the pA-APEX2 experiments but not in the without H_2_O_2_ and IgG controls, the Log_2_ FC values were defined as 100. Proteins with Log_2_ FC > 1 were ultimately detached from background proteins in two independent measurements, which were considered to be potential interacting proteins of corresponding histone marks.

#### Functional gene set enrichment and interaction network visualization

All proteins identified were mapped to mouse Metascape identifiers via gene names. Functional gene set enrichment for each histone mark was performed using the “Custom Analysis” function in Metascape (version 2021_02), with parameters set as follows: overlap ≥ 3, *P* < 0.01, and enrichment score ≥ 1.5. From all enriched proteins in any of the interactions, the top 20 GO cellular component terms (Gene Ontology Consortium, 2020) that were significantly enriched in at least three of the interactors were selected.

STRING (version 11.0) interaction confidences with a confidence score of 0.4 and FDR stringency of 0.05 were added as links between identified proteins. Proteins in vital GO terms with a positive Log_2_ FC in histone mark interactors compared to without H_2_O_2_ and/or IgG were considered to be a visualization foreground. The network of each specific histone mark proximal proteome was imported into Cytoscape (version 3.8.2) and visualized. Cytoscape was used to layout the potential interacting proteins of each histone mark in pA-APEX2 experiments that were members of vital enriched GO terms. Visualization was based on GO term membership.

### Co-immunoprecipitation and Western blotting

In total, 2 × 10^7^ cells were washed with 10 ml PBS, pooled into a 15-ml tube, and centrifuged at 250 *g* for 5 min. The cell pellet was resuspended in 1 ml PBS and crosslinked with 10 ml freshly prepared 1% formaldehyde at room temperature for 10 min, and the reaction was quenched with 1/10 volume of 1.25 M glycine. The tube was inverted several times, shaken gently for 5 min, and centrifuged at 500 *g* for 5 min. The pellet was then washed once with 10 ml ice-cold PBS and centrifuged at 500 *g* for 5 min. Next, the supernatant was carefully aspirated, and the cell pellet was resuspended in 3 ml of cell lysis buffer (10 mM Tris-HCl pH7.5, 10 mM NaCl, 0.5% IGEPAL), and vortexed and incubated on ice for 10 min. The cell pellet was then centrifuged at 3000 r/min for 5 min. The cell pellet was resuspended in 250 μl of MNase digestion buffer [20 mM Tris-HCl pH 7.5, 15 mM NaCl, 60 mM KCl, 1 mM CaCl_2_, MNase (Catalog No. M0247S, NEB)], and incubated at 37 °C for 20 min. Then, 2× Stop ChIP buffer (100 mM Tris-HCl pH 8.1, 20 mM EDTA, 200 mM NaCl, 2% Triton X-100, 0.2% sodium deoxycholate) was added to each tube. Cells were lyzed in RIPA buffer supplemented with protease inhibitor, and the clarified lysates were incubated with 5 µg antibodies overnight at 4 °C. Then, 30 µl protein A/G agarose beads (Catalog No. HY-K0202-5 mL, MCE, Princeton, NJ) were added to the clarified lysates, and the mixture was incubated at 4 °C for 2 h. The beads were then washed three times with 1× ChIP buffer, once with high salt buffer (ChIP buffer plus 0.5 M NaCl), and once with Tris/LiCl buffer (10 mM Tris-HCl pH 8.0, 0.25 M LiCl_2_, 0.5% NP-40, 0.5% sodium deoxycholate, 1 mM EDTA). Samples were then boiled in 5× SDS loading buffer, resolved on SDS-PAGE, and transferred to PVDF membranes. This was followed by blocking with 5% milk in PBST and incubation with the indicated antibodies.

## Data availability

The MS proteomics data have been deposited to the ProteomeXchange Consortium [42] via the PRIDE [43] partner repository (ProteomeXchange: PXD028063), and are publicly accessible at https://www.ebi.ac.uk/pride/.

## CRediT author statement

**Xinran Li:** Methodology, Investigation, Data curation, Writing - original draft. **Jiaqi Zhou:** Software, Formal analysis, Data curation. **Wenjuan Zhao:** Methodology, Validation, Data curation. **Qing Wen:** Methodology, Investigation, Writing - original draft, Funding acquisition. **Weijie Wang:** Validation, Data curation. **Huipai Peng:** Software, Formal analysis. **Yuan Gao:** Writing - review & editing. **Kelly J. Bouchonville:** Writing - review & editing. **Steven M. Offer:** Writing - review & editing, Funding acquisition. **Kuiming Chan:** Writing - review & editing. **Zhiquan Wang:** Conceptualization, Methodology, Writing - original draft, Visualization, Funding acquisition. **Nan Li:** Resources, Methodology, Funding acquisition. **Haiyun Gan:** Conceptualization, Writing - review & editing, Visualization, Supervision, Project administration, Funding acquisition. All authors have read and approved the final manuscript.

## Competing interests

The authors declare no competing interests.

## Supplementary material

Supplementary File S1 **AMAPEX protocol**

Supplementary Table S1 **Identified proximal interactome at H3K9me3, H3K27me3, H3K9me3, H4K5ac, and H4K12ac**

Supplementary Table S2 **Enriched GO cellular components terms of identified proximal interactomes**

Supplementary Table S3 **Identified proximal interactome at H3K27me3 by native APEX2 or HRP methods**

Supplementary Table S4 **Antibody information**
