# Supplementary material for: Defining Proximity Proteome of Histone Modifications by Antibody-mediated Protein A-APEX2 Labeling
Source: Genomics Proteomics Bioinformatics. 2021 Sep 30;20(1):87–100. doi: 10.1016/j.gpb.2021.09.003 (PMC9510856; doi:10.1016/j.gpb.2021.09.003)
Supplement: Supplementary File S1 — AMAPEX protocol [file mmc1.docx]

Protocol: Antibody-mediated protein A-APEX2 labeling (AMAPEX)

Reagent setup

*Apex wash buffer*. Prepare as outlined below. Apex wash buffer can be stored at 4 °C for 1 week.

| Reagent | Final concentration |
| --- | --- |
| HEPES pH 7.5 | 20 mM |
| NaCl | 150 mM |
| Spermidine | 0.5 μM |
| Protease Inhibitor EDTA-Free tablet | 1X |

*Dig-wash buffer*. Prepare as outlined below. Dig-wash buffer can be stored at 4 °C for 2 days.

| Reagent | Final concentration |
| --- | --- |
| Digitonin | 0.05% |
| HEPES pH 7.5 | 20 mM |
| NaCl | 150 mM |
| Spermidine | 0.5 μM |
| Protease Inhibitor EDTA-Free tablet | 1X |

*0.01 Dig-wash buffer*. Prepare as outlined below. Dig-wash buffer can be stored at 4 °C for 2 days.

| Reagent | Final concentration |
| --- | --- |
| Digitonin | 0.01% |
| HEPES pH 7.5 | 20 mM |
| NaCl | 150 mM |
| Spermidine | 0.5 μM |
| Protease Inhibitor EDTA-Free tablet | 1X |

*Antibody buffer*. Prepare as outlined below. Antibody buffer can be stored at 4 °C for 2 days.

| Reagent | Final concentration |
| --- | --- |
| Digitonin | 0.05% |
| HEPES pH 7.5 | 20 mM |
| NaCl | 150 mM |
| Spermidine | 0.5 μM |
| Protease Inhibitor EDTA-Free tablet | 1X |
| EDTA pH 8 | 2 mM |
| BSA | 0.1% |

*2 X Quench buffer*. Prepare as outlined below. Freshly prepare 2 X Quench buffer and kept it on ice.

| Reagent | Final concentration |
| --- | --- |
| Sodium azide | 20 mM |
| Sodium ascorbate | 20 mM |
| Trolox | 10 mM |
| HEPES pH 7.5 | 20 mM |
| NaCl | 150 mM |
| Spermidine | 0.5 μM |
| Protease Inhibitor EDTA-Free tablet | 1X |

▲CRITICAL Solutions used in LC-MS/MS are prepared by LC-MS/MS grade regents including the water.

*RIPA lysis buffer*. Prepare as outlined below. RIPA lysis buffer can be stored at 4 °C for 1 month. Sterilize by filtration. Before use, supplement with 1 mM DTT, 1 mM PMSF and 1× Roche Complete EDTA-free protease inhibitor tablets.

| Reagent | Final concentration |
| --- | --- |
| Tris-HCl pH 7.5 | 50 mM |
| NaCl | 150 mM |
| MgCl_2_ | 1.5 mM |
| EGTA | 1 mM |
| SDS | 1% |
| NP-40 | 1% |
| Sodium deoxycholate | 0.4% |

*Wash buffer*. Prepare as outlined below. Wash buffer can be stored at RT for 1 year. Sterilize by filtration.

| Reagent | Final concentration |
| --- | --- |
| Tris-HCl pH 7.5 | 50 mM |
| SDS | 1% |

*RIPA wash buffer*. Prepare as outlined below. RIPA wash buffer can be stored at 4 °C for 1 month. Sterilize by filtration. Before use, supplement with 1 mM DTT.

| Reagent | Final concentration |
| --- | --- |
| Tris-HCl pH 7.5 | 50 mM |
| NaCl | 150 mM |
| MgCl_2_ | 1.5 mM |
| EGTA | 1 mM |
| SDS | 0.2% |
| NP-40 | 1% |

*8 M Urea buffer*. Prepare as outlined below. Freshly prepare the buffer and keep it at room temperature before use.

| Reagent | Final concentration |
| --- | --- |
| Urea | 8 M |
| Tris-HCl pH 7.5 | 50 mM |

*On-bead digestion buffer*. Prepare as outlined below. On-bead digestion buffer can be stored at RT for 1 month.

| Reagent | Final concentration |
| --- | --- |
| HEPES pH8.0 | 50 mM |
| CaCl2 | 1 μM |
| ACN | 2% |

*Buffer B*. Prepare as outlined below. Buffer B can be stored at RT for 1 month.

| Reagent | Final concentration |
| --- | --- |
| Formic acid | 0.1% |
| Acetonitrile | 50% |

*Buffer A*. Prepare as outlined below. Buffer A can be stored at RT for 1 month.

| Reagent | Final concentration |
| --- | --- |
| Formic acid | 0.1% |

*Buffer C*. Prepare as outlined below. Buffer C can be stored at RT for 1 month.

| Reagent | Final concentration |
| --- | --- |
| Formic acid | 0.1% |
| Acetonitrile | 40% |

Procedure

Culture of MEF cells● Timing 2-3 d

1. MEF cell lines are cultured in DMEM/high glucose supplemented with 10% fetal bovine serum, 100 units/mL penicillin, and 100 mg/mL streptomycin. Mycoplasma testing should be performed every month.
2. Seed ~5 x 10^6^ cells in a 150-mm plate with 20 mL of medium and grow them for 48 h at 37°C under 5% CO_2_.
3. One day before harvesting cells, replace culturing medium with fresh prewarmed medium.

**Harvest and fixation of cells** ● **Timing 2 h**

1. Harvest cells in a 15 ml centrifuge tube at room temperature and count cells. Use ~10^7^ mammalian cells per sample.
2. Centrifuge 5 min 250 x g in a swinging bucket rotor at room temperature and drain the supernatant.
3. Resuspend in 10 mL PBS at room temperature (~10^6^/mL PBS).
4. Drop 37% formaldehyde to a final concentration of 0.1% (e.g., 27 µL to 10 ml) with gently shaking, incubate at room temperature for 10 minutes.
5. Add 1.25 M glycine to quench the cross-linking (e.g., 600 µL to 10 ml).
6. Centrifuge 5 min 600 x g at 4 °C and discard the supernatant by pouring off and inverting onto a paper towel for a few seconds.
7. Resuspend and wash cells once in 10 mL ice cold PBS, centrifuge 5 min for 600 x g at 4 °C, drain the supernatant.
8. Resuspend cells in 1 mL ice cold Apex wash buffer, centrifuge 5 min 600 x g at 4 °C, withdraw liquid and hold on ice.

**Bind primary antibody** ● **Timing 3 h to 1 d**

1. Resuspend cells in 300 µL ice-cold Antibody buffer on ice.
2. Add 3 µL primary antibody to each sample with gentle vortexing.

**▲CRITICAL STEP** Use 1:50-1:100 dilution of the antibodies by default or the manufacturer’s recommended concentration for immunofluorescence.

1. Nutate (or rotate) at room temperature for 2 h or overnight at 4 °C. Liquid should remain in the bottom and on the side of the tube while rocking.

**▲CRITICAL STEP** Negative control (corresponding rabbit or mouse IgG) should be included.

**Bind pA-APEX2** ● **Timing 2 h**

1. Wash cells with 1mL 0.01 dig wash buffer. Centrifuge 5 min 600 x g at 4 °C, withdraw liquid.
2. Mix pA-APEX2 in Antibody buffer to a final concentration of 1:100 for 300 µL per sample.

**▲CRITICAL STEP** pA-APEX2 concentration is 1 μg/μL.

1. Place the tubes on a nutator at room temperature for 1 h.
2. Wash cells with 1mL 0.01 dig wash buffer. Centrifuge 5 min 600 x g at 4 °C and withdraw liquid.
3. Repeat step 18 for twice.

**Biotin labeling** ● **Timing 1 h**

1. Incubate cells with 300 ul Apex wash buffer supplemented with 500 μM biotin- phenol on a nutator at room temperature for 30 min.
2. Add 3 ul of 100 mM H_2_O_2_ in Apex wash buffer for 1 min

**▲CRITICAL STEP** The final concentration of H_2_O_2_ is 1mM. Use freshly prepared H_2_O_2_.

1. Add 300ul 2 x quench buffer to quench the reaction for 5min at room temperature.

**▲CRITICAL STEP** Ensure the final concentration of sodium azide is 10 mM, sodium ascorbate 10 mM and Trolox 5 mM.

1. Wash cells with 1 mL 1 X quench buffer, centrifuge 5 min 600 x g at 4 °C and withdraw liquid and hold on ice.

■PAUSE POINT The cell pellet can be kept at -80 °C for 1 month.

Streptavidin pull-down of biotinylated proteins ● Timing 1 d

1. Lysate cell pellet in RIPA lysis buffer for 15 min on ice.

■PAUSE POINT The cell lysate can be kept at -80 °C for 1 month. Cell lysate can be aliquoted to avoid repeated freezing and thawing.

1. Sonicate the cell lysates at 100 W with 3s on and 3s off for 5 min
2. Boil the cell lysate for 10 min at 100°C to de-crosslink.
3. Clarify the cell lysates by centrifugation, measure the amount of protein in each sample.
4. Save 5% of the supernatant as input for western blot analysis.
5. SDS in the sample was diluted to 0.2% with 1x cold RIPA buffer (without SDS).

▲CRITICAL STEP Reduce the concentration of SDS while the biotinylated protein binds better to Streptavidin–Sepharose beads.

1. Wash Streptavidin–Sepharose beads (GE Healthcare) twice with 1x cold RIPA buffer (0.2% SDS).
2. Incubate the 1mg protein of each sample in 50 µL beads with rotation for 4 h at 4°C.
3. Save 5% of the flow through for later western blot analysis. Wash beads as shown in the in-text table below.

| Times | Buffer | Volume |
| --- | --- | --- |
| Twice | Wash Buffer | 1 mL |
| Twice | RIPA wash buffer | 1 mL |
| Twice | 8 M urea buffer | 1 mL |
| Twice | 30% acetonitrile | 1 mL |
| Twice | 20 mM ammonium bicarbonate | 1 mL |

1. Save 5% of the beads for western blot analysis, and the remaining beads were used for later LC-MS/MS analysis.

Western blot analysis ● Timing 5 h

1. Boil the saved supernatant, flow through and beads in 10 µL 5× protein loading buffer to elute the biotinylated proteins.

■PAUSE POINT The Boiled samples can be kept at -80 °C for 1 month.

1. Run 10% SDS-PAGE using the saved 5% flow through, 5% beads and the eluted biotinylated proteins.
2. Transfer the proteins to 0.22 μm PVDF membrane (Millipore) and stain Ponceau S.
3. Block the blots in 1% BSA in TBST at room temperature for 1 h.
4. Incubate the blots with streptavidin-HRP (Beyotime, A0303, 1:5000 dilution) in TBST for 1 h at room temperature.
5. Wash Blots three times with TBST buffer, 5 min/time.
6. Develop with Clarity Western ECL substrate, and image using a ChemiDoc MP Imaging System (Bio-Rad).

Liquid chromatography mass spectrometry

Mass spectrometry-based proteomic experiments were performed as previously described with minor modifications (Li *et al*, 2013).

On-bead digestion ● Timing 1 d

1. Resuspend the remaining beads with biotinylated protein in 200 µL on-bead digestion buffer supplemented with 10 mM TECP and 40 mM CAA, incubate for 30 min at room temperature.
2. Wash beads with 1 mL on-bead digestion buffer.
3. Resuspend beads in 100 µL on-bead digestion buffer supplemented with 1 µL 0.5 µg LysC (Wako, 125-05061), incubate at 37°C for 3 h.
4. Digest beads in on-bead digestion buffer supplemented with 0.5 µg trypsin (Promega, V5280) at 37°C for 16 h.
5. After the digestion is complete, eluted peptide samples to a fresh tube.

▲CRITICAL STEP Take care to not remove any beads when collecting the supernatant, as they may interfere with downstream analysis.

Desalted and Preparation of the final eluate for LC–MS/MS● Timing 1 d

The peptide samples were desalted using StageTips before LC-MS/MS analysis.

1. Insert C18 material in 200 μL pipette tips to make StageTips.
2. Wash C18 material as shown in the in-text table below.

| Times | Buffer | Volume |
| --- | --- | --- |
| Once | Acetonitrile | 200 μL |
| Once | Buffer B | 200 μL |
| Twice | Buffer A | 1. μL |

1. Load digested peptide samples on stage tips and wash with 100 μL StageTips buffer A twice, and pass the buffer A through.
2. Elute peptide samples as shown in the in-text table below. The solutions were passed through the StageTips by centrifugation at 500 × *g* for 5 min at room temperature.

| Times | Buffer | Volume |
| --- | --- | --- |
| Once | Buffer C | 100 μL |
| Once | Buffer B | 100 μL |

1. Mix the samples in a new Eppendorf tube, and then evaporate ACN in a SpeedVac at 45 °C.
2. lly, all peptides were reconstituted in 0.1% FA (vol/vol) to perform LC-MS/MS analysis.

■PAUSE POINT Prepared peptides can be stored at −80 °C indefinitely.

1. LC-MS/MS data analysis, raw data were processed with MaxQuant (version 1.6.10.43) and its built-in Andromeda search engine for feature extraction, peptide identification, and protein inference. Mouse reference proteome from UniProt Database (UniProtKB/Swiss-Prot and UniProtKB/TrEMBL, version 2020_12) combined with manually annotated contaminant were applied to search the peptides and proteins. The false discovery rate (FDR) values were set to 0.01, and a match-between-runs algorithm was enabled. After searching, the reverse hits, contaminants, and proteins only identified by one site were removed.
