# Supplementary material for: Defining Proximity Proteome of Histone Modifications by Antibody-mediated Protein A-APEX2 Labeling
Source: Genomics Proteomics Bioinformatics. 2021 Sep 30;20(1):87–100. doi: 10.1016/j.gpb.2021.09.003 (PMC9510856; doi:10.1016/j.gpb.2021.09.003)
Supplement: Supplementary Table S4 — Antibody information [file mmc5.docx]

Table S4 Antibody information

| **Antibody** | **Source** | **Identifier** |
| --- | --- | --- |
| H3K4me3 | Active Motif | Cat# 39159; RRID: AB_2615077 |
| H3K9me3 | Abcam | Cat# ab8898; RRID: AB_306848 |
| H3K27me3 | Cell Signaling Technology | Cat# 9733S; RRID: AB_2616029 |
| H4K5ac | Abcam | Cat# ab51997; RRID: AB_2264109 |
| H4K5,8,12ac | Abcam | Cat# ab233193; |
| H4K12ac | Abcam | Cat# ab177793; RRID: AB_2651187 |
| NSD2 | Abcam | Cat# ab75359 |
| IgG | Abcam | Cat# ab6701; RRID: AB_956011 |
| Goat anti-rabbit IgG H&L (HRP) | Abcam | Cat# ab7090 |
| Donkey anti-rabbit IgG (H+L) Alexa Fluor plus 488 | Invitrogen | Cat# A32790; RRID: AB_2762833 |
| Streptavidin−Cy3 | Sigma | Cat# S6402-1ML |
